# Supplementary material for: Microbial quality assessment of Niger seed (Guizotia abyssinica (Linnaeus f.) Cassini) oil in Gondar city: A laboratory-based cross-sectional study
Source: PLoS One. 2025 Sep 9;20(9):e0331546. doi: 10.1371/journal.pone.0331546 (PMC12419661; doi:10.1371/journal.pone.0331546)
Supplement: Attachment — Submitted filename: my review letter.docx [file pone.0331546.s001.docx]

Dear Chief Editor,

I have reviewed the manuscript titled "Microbial Quality Assessment of Niger Seed Oils in Gondar City, Northwest Ethiopia" submitted to PLOS ONE. Overall, the study is well-conducted and presents valuable insights into the microbial quality of Niger seed oils. However, I have identified several issues and questions that need to be addressed before publication.

1) There are some typographic errors in this manuscript which must been corrected.

(line 169 [30 °c, hrs], line 213 [-ml])

2) what is the meaning of C in the line 278 in table2 (370C /48hrs) ? centigrade?
3) The authors have reported that the levels of various microbial contaminations are lower than the standards in Ethiopia, except for fungi. What is the rationale for studying oil contamination after evaluating the standard system? How do the findings contribute to existing knowledge or practices?
4) Considering the recent studies mentioned in the manuscript, what is the novelty of this study? What new insights or contributions does this research provide beyond the existing literature?
5) The manuscript requires a thorough review by a native English speaker to address grammatical errors and improve the coherence of the text.
