# Supplementary material for: Microbial quality assessment of Niger seed (Guizotia abyssinica (Linnaeus f.) Cassini) oil in Gondar city: A laboratory-based cross-sectional study
Source: PLoS One. 2025 Sep 9;20(9):e0331546. doi: 10.1371/journal.pone.0331546 (PMC12419661; doi:10.1371/journal.pone.0331546)
Supplement: Attachment — Submitted filename: Response to reviewers_WAD.docx [file pone.0331546.s002.docx]

**Rebuttal letter**

**Manuscript Title: A review of microbial quality of Niger seed oil in Gondar City: a laboratory-supported cross-sectional study**

**Manuscript ID: PONE-D-24-29805**

Thank you so much for giving us the opportunity to revise the manuscript. We ensured that we addressed all the concerns raised by the editors and reviewers by revising the manuscript thoroughly. Our response to the reviewer’s comment and question is described in detail on the following pages. Furthermore, the details of changes were shown by track changes in the supplementary document attached. Please feel free to contact us again if there are unaddressed issues.

NB. We make an effort to combine and address related concerns that were presented in various portions of the manuscript.

**Comments to the Author**

Reviewers' comments:

Reviewer's Responses to Questions

**Comments to the Author**

1. If the authors have adequately addressed your comments raised in a previous round of review and you feel that this manuscript is now acceptable for publication, you may indicate that here to bypass the “Comments to the Author” section, enter your conflict-of-interest statement in the “Confidential to Editor” section, and submit your "Accept" recommendation.

Reviewer #2: All comments have been addressed

Reviewer #3: (No Response)

**Author’s response: We sincerely thank the reviewers for their thoughtful and constructive feedback throughout the review process. We are grateful that all comments have been thoroughly addressed to your satisfaction.**

2. Is the manuscript technically sound, and do the data support the conclusions?

The manuscript must describe a technically sound piece of scientific research with data that supports the conclusions. Experiments must have been conducted rigorously, with appropriate controls, replication, and sample sizes. The conclusions must be drawn appropriately based on the data presented.

Reviewer #2: Yes

Reviewer #3: Partly

**Author’s response: We thank the reviewers for their careful evaluation of the technical aspects of our manuscript. We are pleased that Reviewer #2 finds the study technically sound and the data supportive of our conclusions. We appreciate Reviewer #3’s partial agreement and would be grateful for any specific concerns or suggestions to further clarify or strengthen the rigor of our experiments.**

3. Has the statistical analysis been performed appropriately and rigorously?

Reviewer #2: Yes

Reviewer #3: No

**Author’s response: We thank the reviewers for their careful consideration of the statistical analysis in our study. We are glad that Reviewer #2 finds the statistical methods appropriate and rigorous. We appreciate Reviewer #3’s concerns and take them seriously. If Reviewer #3 could specify particular aspects of the analysis that require improvement or further explanation, we would be happy to incorporate those suggestions to strengthen the statistical validity of our findings.**

4. Have the authors made all data underlying the findings in their manuscript fully available?

The [PLOS Data policy](http://www.plosone.org/static/policies.action#sharing) requires authors to make all data underlying the findings described in their manuscript fully available without restriction, with rare exception (please refer to the Data Availability Statement in the manuscript PDF file). The data should be provided as part of the manuscript or its supporting information, or deposited to a public repository. For example, in addition to summary statistics, the data points behind means, medians and variance measures should be available. If there are restrictions on publicly sharing data—e.g. participant privacy or use of data from a third party—those must be specified.

Reviewer #2: Yes

Reviewer #3: Yes

**Author’s response: We sincerely thank the reviewers for confirming that all data underlying our findings have been made fully available in accordance with PLOS’s Data Policy.**

5. Is the manuscript presented in an intelligible fashion and written in standard English?

PLOS ONE does not copyedit accepted manuscripts, so the language in submitted articles must be clear, correct, and unambiguous. Any typographical or grammatical errors should be corrected at revision, so please note any specific errors here.

Reviewer #2: Yes

Reviewer #3: Yes

**Author’s response: We sincerely thank the reviewers for their positive assessment of the clarity and quality of the manuscript’s language.**

6. Review Comments to the Author

Please use the space provided to explain your answers to the questions above. You may also include additional comments for the author, including concerns about dual publication, research ethics, or publication ethics. (Please upload your review as an attachment if it exceeds 20,000 characters)

Reviewer #2: Dear Authors,

Thank you for your detailed responses and thoughtful revisions to the manuscript titled "Microbial Quality Assessment of Niger Seed Oils in Gondar City, Northwest Ethiopia." I appreciate the effort you have invested in addressing the concerns raised.

The typographic and formatting issues have been corrected appropriately.

The clarification of "C" in Table 2 is accurate and resolves the query.

The rationale and significance of the study, as well as its contributions to existing knowledge and public health practices, are now well-articulated.

The novelty and specific focus on Niger seed oil are clearly demonstrated, adding substantial value to the literature.

Revisions to improve grammar and coherence have been noted, and I trust these have enhanced the manuscript’s readability.

Overall, your responses comprehensively address the feedback provided. I recommend this revised manuscript for acceptance and publication.

Sincerely,
Reviewer #2

Reviewer #3: The authors of the article "A review of microbial quality of Niger seed oil in Gondar City: a laboratory supported cross-sectional study" described microbial analysis of oil derived from plant Guizotia abyssinica (L.f.) Cass.
In my opinion article requires major corrections:
1. The title should be more informative.

**Author’s response: Thank you for your careful review. Here is our updated article title which will make it more informative “Microbial Quality Assessment of Niger Seed (Guizotia abyssinica (Linnaeus f.) Cassini) Oil in Gondar City: A Laboratory-Based Cross-Sectional Study”.**
2. The whole botanical name of plant should be given.

**Author’s response: Thank you for your careful review. In the current manuscript, we have added the whole botanical name of the plant Niger throughout the manuscript which is “(Guizotia abyssinica (Linnaeus f.) Cassini)”.**
3. The Abstract should be changed; it is divided in 5 parts.

**Author’s response: Thank you for your valuable comment. Currently the abstract is corrected and the headers are removed. In addition, currently, it only contains “Background, Methodology/Principal Findings, and Conclusions/Significance” as per the instruction in the submission guideline of the Journal.**
4. It is unsual to cite Microsoft Excel 2016 in the article (line 39).

**Author’s response: Thank you for your valuable comment. We have removed Microsoft Excel 2016 citation from the article as per the suggestion.**
5. Why is it important to describe data for sesame oil or soybean in this article?

**Author’s response: Thank you for your careful review and valuable question. We totally agree that it is not important to describe data about sesame and soybean oil here, so we deleted it from the text as per the suggestion of the reviewer.**
6. The section Methods should be completely rewritten- why did the authors describe the location and the other characteristic for city Gondar?

**Author’s response: Thank you for your instructive comment and question.** **We have described the location and characteristics of Gondar City in the Methods section to provide essential contextual background that justifies the study setting and enhances the relevance of the findings. Gondar is a key cultural and economic hub in northwest Ethiopia with a substantial population and a notable edible oil market, including numerous wholesalers and producers of both imported and locally produced oils. We thought that detailing the city’s geographic, demographic, and market features helps readers understand the local dynamics affecting edible oil production, distribution, and consumption, which are critical for interpreting microbial contamination risks and quality control challenges specific to this context.**

**But now we have agreed with the suggestion of the reviewer and we only put the description of Gondar’s edible oil market structure-such as the number and types of wholesalers and producers which highlights the complexity and scale of oil supply chains in the area underscoring the importance of evaluating microbial quality in oils marketed there.**
7. What is Kebele (line 141)?

**Author’s response: Thank you for your question. The meaning of Kebele in the manuscript is now defined in bracket as follows “**(smallest administrative unit, essentially a neighborhood or village)**”.**
8. It is unusual to describe all used in laboratory equipment as gloves, Burner bunsen, spatula etc. in research article. It should be changed.

**Author’s response: Thank you for your careful review. It is corrected by removing the detail description of the commonly used laboratory equipment’s as per the suggestion of the reviewer.**
9. Table 3 "Pseudomonas. A; Klebsiella pneumonie" they should be corrected.

**Author’s response: Thank you for your careful review. Now, it is corrected as per the suggestion of the reviewer in the current manuscript.**
10. We have 2 tables 1 and 2, but no table 4 or 5.

**Author’s response: Thank you for your comment. In the revised manuscript, there are six tables in line 271(table 1), line 282(table 2), line292(table 3), lin299(table 4), line306(table 5) and 311(table 6) respectively.**
11. Table 6- many microorganism names written with mistake.

**Author’s response: Thank you for your comment. In the current revised manuscript, all microorganism’s names are corrected as per the suggestion of the reviewer.**
12. line 196 "Diane Roberts provided"- in my opinion citation is enough.

**Author’s response: Thank you for your comment. We have corrected it as suggested by the reviewer.**
13. lines 198-199- the number of plate is in this formula?

**Author’s response: Thank you for your question. Yes, it is to say “number of plates”. We have corrected it from plate – to – plates.**
14. Gram staining is descibed in details.

**Author’s response: Thank you for your comment. It is corrected as per the suggestion of the reviewer.**
15. "Gram staining for fungi"???

**Author’s response: Thank you for your question. We have agreed with comment and changed the heading as follows: “Microscopic Identification of Fungi Using Lactophenol Blue Staining”**
16. Table 2- SD equal to 4x10^4????

**Author’s response: Thank you for your question. The microbial loads for various media were determined, and the results are summarized in Table 2. The mean values represent the average microbial counts, while the standard deviation (SD) indicates the variability within the samples. The standard deviation (SD) quantifies the spread of microbial counts around the mean, reflecting the degree of variability in the samples. For example, the total aerobic mesophilic bacterial count at 37°C showed a mean of 3.4 × 10⁴ CFU with an SD (standard deviation) of 4 × 10⁴, indicating some variability in bacterial populations across replicates.**
17. Line 360-which pathogens did the authors mean?

**Author’s response: Thank you for your question. The pathogens referred are primarily *Staphylococcus aureus* strains, which produce exotoxins capable of causing food poisoning in humans upon consumption, as noted by Levine (1938). Additionally, coliform bacteria, including certain strains of *Escherichia coli* (notably pathogenic strains like E. coli O157:H7), are implicated as potential contaminants posing similar food safety risks.**
