# Supplementary material for: Microbial quality assessment of Niger seed (Guizotia abyssinica (Linnaeus f.) Cassini) oil in Gondar city: A laboratory-based cross-sectional study
Source: PLoS One. 2025 Sep 9;20(9):e0331546. doi: 10.1371/journal.pone.0331546 (PMC12419661; doi:10.1371/journal.pone.0331546)
Supplement: Attachment — Submitted filename: Response to Reviewers.KDM.docx [file pone.0331546.s003.docx]

**Rebuttal letter	8/17/2025**

**Manuscript Title: Microbial Quality Assessment of Niger Seed (Guizotia abyssinica (Linnaeus f.) Cassini) Oil in Gondar City: A Laboratory-Based Cross-Sectional Study**

**Manuscript ID: PONE-D-24-29805**

Thank you so much for giving us the opportunity to revise the manuscript again. We ensured that we addressed all the concerns raised by the editors and reviewers by revising the manuscript thoroughly. Our response to the reviewer’s comment and question is described in detail on the following pages. Furthermore, the details of changes were shown by track changes in the supplementary document attached. Please feel free to contact us again if there are unaddressed issues.

NB. We make an effort to combine and address related concerns that were presented in various portions of the manuscript.

**Comments to the Author**

Reviewers' comments:

Additional Editor Comments (if provided):

Please, authors, thank you for your patience. As you can see, reviewers still have some concerns. Kindly make an effort to address the concerns where you can.
For example, condense/shorten the step-by-step methodology for Gram staining, as well as "A microscopic method for identifying fungi"—
In the Materials section, delete the list of volumetric flasks, Petri dishes, glass rods, etc., because these are standard laboratory equipment.
In the section, Data quality assurance and control methods, further condense and shorten it.
Please, address all other concerns raised by the other reviewer.
Looking forward to your revised manuscript.

**Author's response: Thank you very much dear reviewer for your thorough review and constructive feedback. We truly appreciate your patience and thoughtful suggestions.**

**We have carefully considered all the concerns raised, and we are committed to improving the manuscript accordingly. Specifically:**

- **The step-by-step methodology for Gram staining and the microscopic method for identifying fungi have been carefully condensed and shortened for clarity and conciseness.**

**In the Materials section, we have removed the detailed list of standard laboratory equipment (volumetric flasks, Petri dishes, glass rods, etc.) to streamline the presentation and focus on key materials.**

- **The data quality assurance and control methods section is now deleted as per the suggestion of the reviewer.**
- **We have thoroughly addressed all other points raised by the reviewers to the best of our ability.**

**We believe these revisions have improved the manuscript significantly and look forward to your continued guidance.**
Reviewers' comments:

Reviewer's Responses to Questions

**Comments to the Author**

1. If the authors have adequately addressed your comments raised in a previous round of review and you feel that this manuscript is now acceptable for publication, you may indicate that here to bypass the “Comments to the Author” section, enter your conflict-of-interest statement in the “Confidential to Editor” section, and submit your "Accept" recommendation.

Reviewer #3: (No Response)

Reviewer #4: All comments have been addressed

**Author’s response: Thank you very much for your time and thoughtful review of our manuscript. We are pleased to hear that all your comments and concerns have been adequately addressed. We appreciate your constructive feedback, which greatly contributed to improving the quality of our work.**

2. Is the manuscript technically sound, and do the data support the conclusions?

The manuscript must describe a technically sound piece of scientific research with data that supports the conclusions. Experiments must have been conducted rigorously, with appropriate controls, replication, and sample sizes. The conclusions must be drawn appropriately based on the data presented.

Reviewer #3: Partly

Reviewer #4: Yes

**Author’s response: We thank the reviewers for their careful evaluation of the technical aspects of our manuscript. We appreciate Reviewer #4’s positive assessment that the study is technically sound and that the data support the conclusions. Regarding Reviewer #3’s comment indicating a partial agreement, we would appreciate further clarification to address any specific concerns they may have.**

**In our manuscript, we have taken great care to ensure that all experiments were conducted rigorously with appropriate controls and adequate replication as detailed in the methods section. Statistical analyses were performed to validate the robustness of the findings. The conclusions were drawn directly based on the presented data, avoiding overstated interpretations.**

**If Reviewer #3’s concerns relate to specific experiments or data points, we are happy to provide additional analyses or clarification as needed to strengthen the technical rigor and data support. We are committed to ensuring that the manuscript meets the highest scientific standards and welcome suggestions to improve the clarity and robustness of our study.**

3. Has the statistical analysis been performed appropriately and rigorously?

Reviewer #3: I Don't Know

Reviewer #4: Yes

**Author’s response: We thank the reviewers for their evaluation of the statistical analysis employed in our study. We appreciate Reviewer #4’s confirmation that the statistical methods were appropriate and rigorous. Regarding Reviewer #3’s indication of uncertainty, we would like to clarify that the data were carefully managed and analyzed using Stata Version 14. Descriptive statistics, including means and standard deviations, were computed to summarize microbial counts across samples. Where relevant, serial dilution and colony counting followed standard microbiological quantification protocols to ensure accuracy.**

**Due to the cross-sectional nature and the descriptive objectives of the study, advanced inferential statistical tests were not deemed necessary. However, if Reviewer #3 seeks additional analysis, such as comparisons across sampling sites or brands, or statistical tests to assess variability, we are willing to provide these upon request. Our primary aim was to reliably characterize the microbial quality of available Niger seed oil products, and we believe the current analysis delivers valid and reproducible results that support our conclusions.**

**Please let us know if further detail or additional analyses would be helpful.**

4. Have the authors made all data underlying the findings in their manuscript fully available?

The [PLOS Data policy](http://track.editorialmanager.com/CL0/http:%2F%2Fwww.plosone.org%2Fstatic%2Fpolicies.action%23sharing/1/010f019888eec104-13787630-5ad5-4a6c-807f-dd974a5054b5-000000/qls_zkvsRyvywItzjVUsP7g7NpUwRS95l-cZ-yfBVHc=223) requires authors to make all data underlying the findings described in their manuscript fully available without restriction, with rare exception (please refer to the Data Availability Statement in the manuscript PDF file). The data should be provided as part of the manuscript or its supporting information, or deposited to a public repository. For example, in addition to summary statistics, the data points behind means, medians and variance measures should be available. If there are restrictions on publicly sharing data—e.g. participant privacy or use of data from a third party—those must be specified.

Reviewer #3: Yes

Reviewer #4: Yes

**Author’s response: We appreciate the reviewers’ positive assessment regarding the availability of data underlying our findings. In accordance with the PLOS data policy, we confirm that all data supporting the conclusions of this study are fully available upon request. We are committed to transparency and reproducibility.**

5. Is the manuscript presented in an intelligible fashion and written in standard English?

PLOS ONE does not copyedit accepted manuscripts, so the language in submitted articles must be clear, correct, and unambiguous. Any typographical or grammatical errors should be corrected at revision, so please note any specific errors here.

Reviewer #3: Yes

Reviewer #4: Yes

**Author’s response:** **We sincerely thank the reviewers for their positive evaluation of the manuscript’s clarity and presentation. We have made every effort to ensure that the manuscript is written in clear, correct, and standard English suitable for publication.**

6. Review Comments to the Author

Please use the space provided to explain your answers to the questions above. You may also include additional comments for the author, including concerns about dual publication, research ethics, or publication ethics. (Please upload your review as an attachment if it exceeds 20,000 characters)

Reviewer #3: The authors of the article made a lot of corrections, but in my opinion the work still does not meet the usual standards set for published articles. For example the authors describe methodology for Gram staining step by step; everyone knows how to do it. The same applies to the section "A microscopic method for identifying fungi"- there is a step by step recipe for how to do it.

**Author’s response: We sincerely thank Reviewer #3 for the constructive feedback and for acknowledging the corrections made in the manuscript. We appreciate the valuable perspective regarding the level of detail provided in certain methodological and materials descriptions.**

**Our intention in including step-by-step protocols for procedures such as Gram staining and microscopic fungal identification was to ensure full transparency and reproducibility, particularly given the interdisciplinary readership of the journal. While these methods are indeed standard in many microbiology laboratories, we aimed to provide sufficient detail so that readers from diverse backgrounds, including those less familiar with routine microbiological techniques, could fully understand the processes employed in this study. However, we understand the reviewer’s suggestion and totally agree to remove it in the revised manuscript.**

In the Materials section- the authors list: volumetric flasks, Petri dishes, glass rod etc. Why to do it? It is a standard equipment for every laboratory.

**Author’s response: We sincerely thank you for the constructive comment and question. We included a list of common laboratory equipment to emphasize the rigor and thoroughness of the sample processing environment and to affirm that all materials conformed to standard sterile laboratory practice. However, we understand the reviewer’s suggestion that some of this detail may be unnecessarily extensive for the intended audience. Accordingly, we propose to condense these sections by removing overly detailed stepwise descriptions and standard equipment listings.**

The section Data quality assurance and control methods- should be omitted.

**Author’s response: We thank you dear reviewer, for the helpful suggestion regarding the "Data quality assurance and control methods" section. We agree that omitting this section will streamline the manuscript and improve its overall clarity and readability. Finally, we have removed it from the revised manuscript.**

Reviewer #4: The authors on this manuscript "Microbial Quality Assessment of Niger Seed (Guizotia abyssinica (Linnaeus f.) Cassini) Oil in Gondar City: A Laboratory-Based Cross-Sectional Study," which apparently appraised or revealed Ethiopia's lack of stringent quality control and regulatory oversight, further raising concerns about public health and safety.
They further opined, from their result, that the oil processing, production, handling, and storage systems seemed not to have proper hygienic handling practices.
On careful scrutiny, I could see that the previous reviewers' queries had been addressed, although no table should be allowed to crisscross pages (take note of Table 1, pp. 11-12, and Table 3, pp. 12-13).

Summarily, if the editors are satisfied with the response to the reviewers queries, decision could be made.

**Author’s response: We sincerely thank Reviewer #4 for the thorough and positive evaluation of our manuscript and for acknowledging that the reviewers’ queries have been appropriately addressed. We appreciate the insightful summary regarding the public health implications of our findings related to the hygiene practices in Niger seed oil processing and handling.**

**We also appreciate the constructive comment regarding the formatting of tables. We will carefully revise Tables 1 and 3 to ensure that they are presented fully on a single page, avoiding any breaks across pages, to enhance the manuscript’s readability and presentation quality. Thank you again for your valuable feedback and support for the consideration of our manuscript.**

**Additional changes to the revised manuscript**

1. We have included the strengths and limitations of the study.
2. We have outlined the implications of the study.
3. We have also incorporated a map of the study area into the manuscript.
